# Efficacy and safety of thyroxine therapy on patients with heart failure and subclinical hypothyroidism: A protocol for systematic review and meta-analysis

**DOI:** 10.1097/MD.0000000000023947

**Published:** 2021-01-22

**Authors:** Hongshuo Shi, Zunqi Kan, Yufan Liu, Wenwen Li, Min Peng, Tiantian Yang

**Affiliations:** a Shandong University of Traditional Chinese Medicine; b Department of Traditional Chinese Medicine, Shandong Provincial Hospital Affiliated to Shandong First Medical University, Jinan, ShanDong, China.

**Keywords:** heart failure, meta-analysis, subclinical hypothyroidism, thyroxine

## Abstract

**Background:**

Subclinical hypothyroidism (SCH) can increase the risk of heart failure (HF) clinically. However, thyroxine therapy for patients with HF and SCH has the risk of developing tachyarrhythmias. At present, there is no sufficient evidence-based medical evidence for levothyroxine in the therapy of this situation, and the treatment issue is still controversial. Therefore, our meta-analysis aims to assess the effectiveness and safety of thyroxine therapy for patients with HF and SCH.

**Methods:**

We searched the related randomized controlled trials that have been published in the following 7 electronic databases: PubMed, Cochrane Library, EMBASE, Chongqing VIP, China National Knowledge Infrastructure, Chinese biomedical literature database, and Wan Fang database. The treatment group was treated with routine HF therapy plus thyroxine, while the control group was treated with HF routine therapy. Main outcome measures effective rate and New York Heart Association classification; Secondary outcome measures included: left ventricular ejection fraction, quality of life score, brain natriuretic peptide / N-terminal pro brain natriuretic peptide, 6-minute walk test, and adverse events. After screening studies and extracting data, we will use Cochrane collaborative tools to evaluate the risk of bias to assess the methodological quality of the included randomized controlled trials. We will use STATA 14.0 software for data synthesis and statistical analysis. Both subgroup analysis and sensitivity analysis will be used to detect potential sources of heterogeneity. In addition, we will use sensitivity analysis to test the stability of the outcomes. If possible, we will perform a funnel chart and Eggers test evaluate publication bias. The quality of the evidence will be evaluated through the grades of recommendations assessment, development, and evaluation system.

**Results:**

Our findings will be published in peer-reviewed journals.

**Conclusion:**

This research will provide evidence about the efficacy and safety of thyroxine in the treatment of patients with HF and SCH. Objective to provide evidence-based medicine basis for thyroxine treatment of patients with SCH and HF.

**Registration number:**

INPLASY2020100062.

## 1 Introduction

Heart failure (HF) is caused by cardiovascular diseases caused by multiple reasons, leading to a series of systolic and diastolic dysfunction, usually mediated by different ventricular remodelling patterns.^[1]^ Although the mortality rate of cardiovascular disease (CVD) has decreased overall, HF is the only major CVD whose prevalence and morbidity are thought to be increasing.^[2]^ As an epidemic, HF affected nearly 40 million people worldwide. The Rotterdam study estimates that the prevalence of the disease in the general population is about 2%, and it rises to 17.4% in people ≥85 years old,^[1]^ and the long-term prognosis associated with HF is poor. HF starts with changes in ventricular, diastolic, and/or systolic functions, but then produces and involves changes in biochemistry, metabolism, hormones, and neurohormones.^[3]^ The poor prognosis of HF is partly due to the effects of comorbidities, including changes in thyroid function.

Subclinical hypothyroidism (SCH) is a typical asymptomatic state, biochemically defined as elevated serum TSH concentration and normal free T4 (FT4) levels.^[4]^ More and more studies have shown that both SCH and subclinical hyperthyroidism have profound effects on heart function by regulating systolic and diastolic function, heart rate, and systemic vascular resistance.^[5]^ A meta-analysis of a large prospective cohort showed that compared with normal thyroid function, the risk of HF events in both SCH and hyperthyroidism was increased.^[6]^ It is not clear whether levothyroxine has any benefit in preventing cardiovascular events in patients with SCH, and controversy over the need for treatment still prevails,^[7]^ and levothyroxine therapy in patients with CVD and SCH is at risk of developing tachyarrhythmia.^[8]^ Meta-analysis is a dependable method that can resolve differences in research. However, there is currently no meta-analysis on the efficacy and safety of thyroxine in the therapy of patients with HF and SCH. In this meta-analysis, we combined the relevant randomized controlled trials (RCTs) of thyroxine treatment in patients with HF and SCH to clarify the efficacy and safety of thyroxine treatment.

## 2 Methods

### 2.1 Research registration

Our meta-analysis will be guided by the 2015 Preferred Reporting Items for Systematic Reviews and Meta-Analysis-P preferred reporting project.^[9]^

We have registered our protocol on the INPLASY website with the number of INPLASY2020100062 (https://inplasy.com/).

### 2.2 Eligibility criteria

#### 2.2.1 Participant

(1) Age ≥ 18;
(2) Patients diagnosed with HF and SCH according to any of the diagnostic criteria are eligible to be included;
(3) There are no restrictions on race, nationality, gender, or age;
(4) Before inclusion in the study, patients were not treated with thyroxine;
(5) Patients with cardiac resynchronization therapy or coronary artery bypass surgery, or with severe non-cardiovascular events were excluded.

#### 2.2.2 Interventions and comparators

The treatment group was treated with routine HF therapy plus thyroxine, while the control group was treated with HF routine therapy. The routine therapy in each study may not be the same, but treatment with thyroxine is the only difference between intervention and control.

#### 2.2.3 Outcomes

Main outcome measures effective rate and New York Heart Association classification ; Secondary outcome measures included: left ventricular ejection fraction , quality of life score, brain natriuretic peptide / N-terminal pro brain natriuretic peptide, 6-minute walk test, and adverse events such as rash or itchy skin, dizziness, nausea, vomiting, dry cough, etc.

#### 2.2.4 Study design

We will include RCTs for meta-analysis. At the same time, we will exclude the same studies, reviews, letters, abstracts, or animal experiments.

### 2.3 Study search

We searched PubMed, Cochrane Library, EMBASE, Chongqing VIP, China National Knowledge Infrastructure, Chinese biomedical literature database, and Wan Fang database from the establishment to November 15, 2020 related documents. We use the search strategy of MeSH terms combined with free-text. The MeSH terms we used in this study are: “Thyroxine’,” “Heart Failure’,” “Hypothyroidism,” and “Subclinical (Table 1).” Then, we will use EndNote software to screen the retrieved literature. We do not set language restrictions on the searched documents. In addition, we also screened the references of the retrieved trials or reviews to supplement our included literature.

**Table 1:** Search strategy of PubMed.

Number	Search terms
#1:	(Subclinical[MeSH])
#2:	(Hypothyroidism[MeSH])
#3:	(Hypothyroidisms) OR ("Primary Hypothyroidism")OR ("Hypothyroidism, Primary") OR ("Primary Hypothyroidisms") OR ("Thyroid-Stimulating Hormone Deficiency") OR ("Deficiency, Thyroid-Stimulating Hormone") OR ("Hormone Deficiency, Thyroid-Stimulating") OR ("Thyroid Stimulating Hormone Deficiency") OR ("Thyroid-Stimulating Hormone Deficiencies") OR ("TSH Deficiency") OR ("Deficiency, TSH") OR ("TSH Deficiencies") OR ("Deficiency, TSH") OR ("TSH Deficiencies") OR ("Secondary Hypothyroidism") OR ("Hypothyroidism, Secondary") OR ("Secondary Hypothyroidisms") OR ("Central Hypothyroidism") OR ("Central Hypothyroidisms") OR ("Hypothyroidism, Central")
#4:	#2 OR #3
#5:	#1 AND #4
#6:	(Thyroxine[MeSH])
#7:	("O-(4-Hydroxy-3,5-diiodophenyl)-3,5-diiodotyrosine") OR ("T4 Thyroid Hormone") OR ("3,5,3’,5’-Tetraiodothyronine") OR (Thyroxin) OR ("Thyroid Hormone, T4") OR (Synthrox) OR ("Levothyroxine Sodium") OR ("Sodium Levothyroxine") OR (Thyrax) OR (Tiroidine) OR ("Tiroxina Leo") OR (Unithroid) OR (Eferox) OR (Eltroxin) OR (Thevier) OR (Eltroxine) OR (Euthyrox) OR (Eutirox) OR ("L-Thyrox") OR ("L Thyrox") OR ("L-Thyroxin beta") OR ("L Thyroxin beta") OR ("L-Thyroxin Henning") OR ("L Thyroxin Henning") OR (Levothyroxine) OR ("O-(4-Hydroxy-3,5-diiodophenyl) 3,5-diiodo-L-tyrosine") OR ("L-Thyroxine") OR ("L Thyroxine") OR ("L-3,5,3’,5’-Tetraiodothyronine") OR (Levoxine) OR (Levoxyl) OR (Lévothyrox) OR ("L-Thyroxine Roche") OR ("L Thyroxine Roche") OR ("Levo-T") OR ("Levo T") OR (Levothroid) OR (Novothyral) OR (Berlthyrox) OR (Dexnon) OR (Novothyrox) OR (Oroxine) OR (Synthroid) OR ("Levothyroxin Deladande") OR ("Levothyroxin Delalande") OR (Levothyroid)
#8:	#6 OR #7
#9:	("Heart Failure"[Mesh])
#10:	("Heart Decompensation") OR ("Decompensation, Heart") OR ("Heart Failure, Right-Sided") OR ("Heart Failure, Right Sided") OR ("Right-Sided Heart Failure") OR ("Right Sided Heart Failure")OR ("Myocardial Failure") OR ("Congestive Heart Failure") OR ("Heart Failure, Congestive") OR ("Heart Failure, Left-Sided") OR ("Heart Failure, Left Sided") OR ("Left-Sided Heart Failure") OR ("Left Sided Heart Failure") OR ("Cardiac Failure")
#11:	#9 AND #10
#12:	#5 AND #8 AND #11

### 2.4 Data collection and analysis

#### 2.4.1 Selection of researches

**Figure 1:**
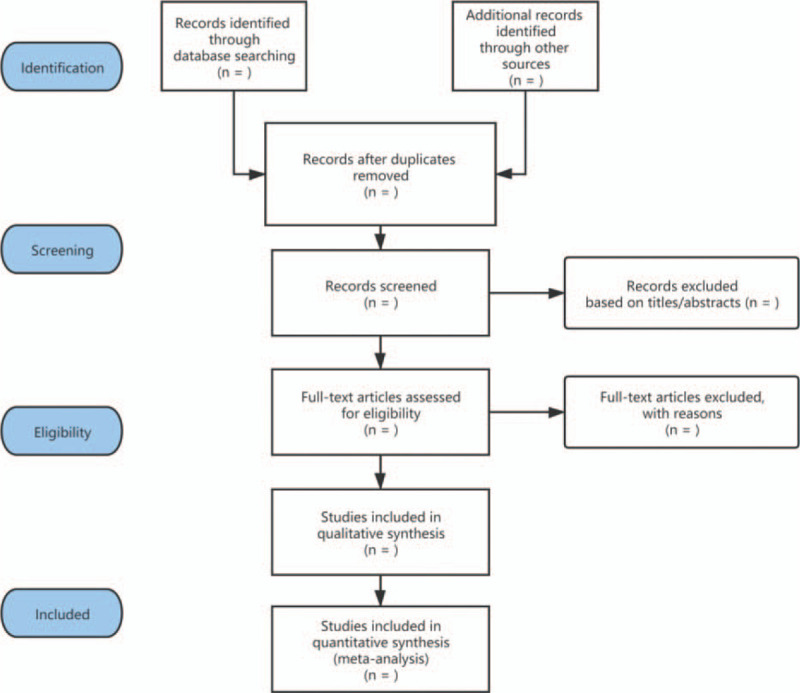
The flowchart of the screening process.

We use EndNote software to screen the retrieved related literature. After we exclude duplicate documents, 2 independent researchers will read the titles and abstracts of all the literature for preliminary screening, and then read the full text carefully before deciding whether to include them. If there is any objection in the process of literature screening, the scheme will be submitted to the third party for negotiation. In addition, our screening process is shown in Figure 1.

#### 2.4.2 Data extraction and saving

Two independent researchers will produce Microsoft Excel to extract and manage relevant clinical data eventually included in the literature. We are going to extract the following clinical information: title, first author, sample size, year, included population, age, gender, intervention measures, disease course, results, and adverse reactions. If the clinical data of the relevant studies in the literature are insufficient, we will try to contact the relevant authors for integrated clinical information. If we are not able to contact the author, due to a lack of important information, we will exclude the study.

### 2.5 Risk of bias assessment

All included studies will be assessed by the guidelines in the Cochrane Handbook. We will assess the inclusion of the study from the following 7 projects. They are random sequence generation, allocation hiding, participants and people blindness, results evaluation blind, results data incomplete, selective results report, and other deviations. The quality of each randomized controlled trials is classified as ”high,” “low” or “unclear”^[10]^ risk of bias. When there are differences, we will reach a consensus through discussions with third parties.

### 2.6 Data analysis

We will use Stata 14.0 software to conduct a meta-analysis of the included studies. Binary variables use relative risk and 95% CI as the statistical effect size. When continuous variables have the same measurement unit, they are expressed as weighted mean difference with 95% CI. When the measurement unit is different, use standardized mean difference. with 95% CI. χ^2^ was used for the heterogeneity test. When I^2^<50%, the fixed-effects model was used for meta-analysis; when I^2^>50%, the random-effects model was used for meta-analysis.

### 2.7 Subgroup analysis

We will make subgroup analysis according to age, gender, TSH level, intervention time, drug dosage, and other reasons, subgroup analysis is also an effective method to explore the source of heterogeneity.

### 2.8 Sensitivity analysis

In order to determine the robustness of the results, we will conduct a sensitivity analysis. We will exclude each study in turn, then recombine the analysis data and compare the differences between the results and the original results. If there is significant heterogeneity in the study, we will also use this method to detect whether a study has caused significant heterogeneity.

### 2.9 Publication bias assessment

If we include more than 10 studies, we will use funnel plot and egger regression to assess publication bias and present the results.^[11]^

### 2.10 Grading the quality of evidence

We will use the grades of recommendations assessment tool to evaluate the quality of evidence and grade the results. grades of recommendations assessment tool is a widely used tool to evaluate the quality of evidence. The assessment will be divided into high quality, medium quality, low quality, and very low quality.

### 2.11 Included population participation

The systematic review and meta-analysis did not include the relevant population.

### 2.12 Ethics and dissemination

Our study is a secondary study based on RCTs and belongs to systematic review and meta-analysis. Therefore, ethical approval is not required. Our study results will also be published in peer-reviewed relevant journals and reported at relevant meetings.

## 3 Discussion

HF is the terminal stage of almost all forms of heart disease and is one of the most common causes of hospitalization and death worldwide.^[12,13]^ The poor prognosis of HF is partly due to the effects of comorbidities including changes in thyroid function.^[14–16]^ The effect of thyroxine on cardiovascular events has not been fully studied, and treatment issues are still controversial. Because it is concerned about the potentially harmful effects of thyroxine treatment, current guidelines recommend that patients with CVD and SCH should start taking low doses of levothyroxine compared with SCH without cardiac complications.^[17]^ Some studies have already produced results. Once SCH patients are treated with levothyroxine, their physical fitness measured by a 6-minute walk is significantly improved,^[18]^ and another study also showed that levothyroxine treatment can optimize the treatment of HF patients with systolic left ventricular dysfunction and SCH.^[19]^ However, studies have also shown that compared with placebo, elderly people with mild SCH have no difference in systolic and diastolic heart function after levothyroxine treatment.^[19]^ The contradictions of these studies make our meta-analysis more meaningful.

### 3.1 Amendments

If our meta-analysis process needs to be modified, we will correct our proposal in time.

## Author contributions

The protocol was designed by SHS and YTT. All the authors participated in the study. The manuscript was drafted by PM and LYF and revised by LWW and KZQ. YTT will be responsible for the study. All authors approved the final manuscript before submission.

**Data curation:** Yufan Liu, Min Peng.

**Formal analysis:** Wenwen Li, Min Peng.

**Funding acquisition:** Tiantian Yang.

**Methodology:** Hongshuo Shi, Tiantian Yang.

**Project administration:** Tiantian Yang.

**Software:** Zunqi Kan.

**Writing – original draft:** Hongshuo Shi, Tiantian Yang.

**Writing – review & editing:** Yufan Liu, Wenwen Li, Tiantian Yang.
